# The association between physical activity, self-efficacy, stress self-management and mental health among adolescents

**DOI:** 10.1038/s41598-024-56149-4

**Published:** 2024-03-06

**Authors:** Ge Zhang, Wanxuan Feng, Liangyu Zhao, Xiuhan Zhao, Tuojian Li

**Affiliations:** School of Physical Education, Shandong University, Jinan, 250061 China

**Keywords:** Physical activity, General self-efficacy, Stress self-management, Mental health, Physical education, Psychology, Risk factors

## Abstract

This study aimed to explore the interplay between physical activity, self-efficacy, stress self-management and mental health among adolescents. The study gathered data from an online survey conducted among 400 Chinese middle school students (mean age = 13.74 years). The collected data were analyzed using SPSS 27.0 and PROCESS 4.1. The findings indicated a positive and significant relationship between physical activity, self-efficacy, stress self-management, and mental health. Notably, the association between physical activity and mental health was entirely mediated by self-efficacy and stress self-management. Moreover, self-efficacy and stress self-management exhibited a chain mediation effect on the relationship between physical activity and mental health. It is suggested that interventions focusing on physical activity should prioritize strategies for enhancing students’ self-efficacy and stress self-management skills as integral components of promoting adolescents’ mental health. Future research should delve into identifying specific types of physical activities that have a greater potential to enhance self-efficacy and stress self-management abilities.

## Introduction

According to the WHO, 14% 10–19 year-olds experience mental health conditions, accounting for 13% of the global burden of disease in this age group. Depression, anxiety and behavioural disorders are among the leading causes of illness and disability among adolescents. By 2030, depression alone is projected to become the primary cause of disability and reduced life expectancy^1^. Adolescents are immature in physiological and psychological development and facing greater pressures in various aspects such as higher education, self and peer, are observed to be more susceptible to experiencing mental health issues^2,3^. Moreover, psychological challenges during adolescence can significantly influence mental health in adulthood, leading to adverse outcomes^4^. Understanding the pathways that contribute to adolescents’ stress self-management levels and mental health is crucial for implementing effective measures to prevent and alleviate serious issues like anxiety and depression.

Physical activity is recognized as a significant means to foster the holistic development of adolescents, encompassing both physical and mental health. Prior studies have indicated that engaging in moderate-to-vigorous physical activity is linked to reduced depression and anxiety^5,6^. Consistently participating in physical activity has been correlated with improved mental health^7–9^. Regular engagement in physical activity implies that adolescents develop better self-management skills and time allocation, often characterized by increased autonomy. This behavior reduces the likelihood of engaging in activities detrimental to their mental well-being, such as excessive screen time^10^. In addition, they can cope with the emergency stressors better which makes them keep more stable mental health^11^. However, a recent study discovered that adolescents engaging exclusively in individual physical activities reported higher psychological issues like anxiety and depression compared to non-participants^12^. Furthermore, a study demonstrated that individuals with high rumination stress exhibited heightened negative emotional responses after a solitary riding intervention subsequent to stress-inducing stimuli^13^. Variations in the type of physical activity appear to yield distinct effects on mental health, with individuals’ characteristics also influencing how physical activity impacts their mental well-being^14^. The precise psychological processes altered by physical activity remain to be clearly delineated.

Self-efficacy, a key component of Bandura’s social cognitive theory, is considered integral to social competence. It encompasses individuals’ belief in their ability to effectively navigate the demands of dynamic societal conditions and to tackle challenges in evolving societies^15^. Connolly discovered a connection between one’s behavior, social environment, and self-efficacy^16^. Furthermore, the exercise and self-esteem models suggest that physical activity is linked to the process of self-perception and self-evaluation. Individuals who engage in regular physical activity consistently demonstrate elevated levels of self-efficacy^17^. Furthermore, a previous study indicated that individuals with initially high levels of physical activity, even if declining, exhibited greater self-efficacy than those with low physical activity levels that were also declining^18^. This phenomenon could be attributed to the consistent accomplishment of self-set physical activity goals, contributing to heightened self-confidence and self-perception. Consequently, individuals may develop an enhanced sense of self-efficacy. Elevated levels of self-efficacy correlate with reduced depression and anxiety, as well as an increased sense of subjective well-being^19^. Earlier investigations demonstrated that typically developing adolescents exhibited higher self-efficacy in contrast to emotionally disturbed counterparts^16^. Individuals with greater self-efficacy exhibit increased confidence in addressing unforeseen life challenges, approach problems with a positive attitude, thereby fostering a heightened stability in their mental health. Physical activity potentially enhances mental well-being via the intermediary of self-efficacy, achieved by heightening the pleasure and emotional states during acute exercise sessions^20^.

Stress management involves techniques aimed at helping individuals recognize and cope with stressors through a range of strategies^21^. In contrast to general stress management, stress self-management pertains specifically to stress management behaviors undertaken voluntarily by individuals^22^. Earlier research categorized stress management strategies into dimensions of problem-focused, emotion-focused, and avoidance strategies^23^. Optimal selection of stress management strategies appears contingent upon the nature of the stressor. Broadly, problem-focused and emotion-focused strategies are adaptive coping mechanisms, while avoidance strategies tend to be maladaptive^23–25^. Individuals capable of selecting adaptive stress management strategies for particular stressors demonstrate enhanced stress self-management skills. Currently, adolescents primarily contend with substantial stressors arising from academic demands, which have exhibited a gradual upward trend over the past two decades^26^. Physical activity is recognized as a crucial stress management strategy. Research indicates that individuals engaging in regular physical activity exhibit improved cognitive and executive functions^27,28^. They are also more inclined to employ adaptive coping strategies for daily stressors and demonstrate enhanced resilience in facing unexpected challenges. Regular participation in physical activity is typically linked to elevated levels of stress self-management. Hampel’s research revealed that adolescents often employ maladaptive stress self-management strategies to address life stressors, interestingly, adaptive problem-solving and emotion-focused stress management strategies exhibited a decline with advancing age^29^. Research suggests that adolescents who predominantly resort to maladaptive stress self-management strategies are at a heightened risk of developing depression and anxiety^30^. Prioritizing stress management that centers on addressing the stressor directly for problem resolution, rather than avoiding or redirecting attention has been linked to improved mental health^31^. Consistent engagement in physical activity is believed to enhance cognitive and executive capacities in adolescents^27^. Consequently, adolescents acquire improved stress management skills, enabling them to address stressors directly and subsequently fostering positive impacts on mental health.

Self-efficacy and stress self-management may mediate the association between physical activity and mental health, but there is also an association between them. Drawing from self-efficacy theory, individuals tend to steer clear of tasks and situations they perceive as exceeding their capabilities, while engaging in activities they believe they are competent at^32^. Individuals with higher self-efficacy possess greater confidence in their abilities, leading them to embrace adaptive stress self-management strategies instead of avoidance tactics. As a result, they generally exhibit enhanced stress self-management skills. Prior studies have demonstrated that individuals with elevated self-efficacy are more inclined to employ constructive stress coping strategies^29,33^. Collectively, the mentioned studies reveal that self-efficacy and stress self-management, integral aspects of self-perception and self-competence, are regarded as significant protective factors for mental health^19,31^. It appears that the link between physical activity and mental health might be influenced initially by self-efficacy and subsequently by stress self-management.

**Figure 1:**
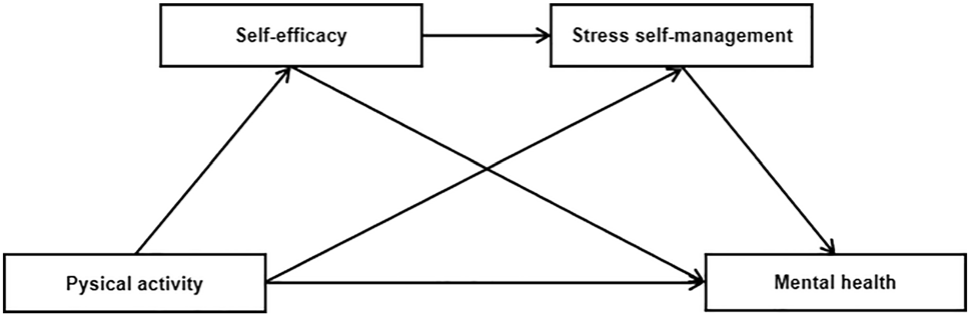
Hypothesis model.

Despite frequent exploration of the association between physical activity and mental health, the underlying mechanisms of this relationship largely remain elusive. Specifically, uncertainties persist regarding the impact of regular participation in physical activity on stress self-management ability, and whether self-efficacy and stress self-management act as mediators in the connection between physical activity and mental health. Consequently, there is a compelling need to investigate these interrelationships among adolescents. Furthermore, adolescents encounter stressors from various dimensions, and these stressors stand as significant contributors to psychological issues. Exploring protective factors influencing adolescents’ stress self-management levels and mental health statuses is pivotal for effective interventions aimed at enhancing adolescent mental well-being. Hence, the present study aimed to explore the correlation between physical activity, self-efficacy, stress self-management, and mental health among adolescents. Furthermore, it seeks to delve into the potential mediating roles of self-efficacy and stress self-management. Drawing from previous research, we have formulated a theoretical model (Fig. 1) and put forth the following hypotheses: (1) physical activity is positive related to mental health. (2) self-efficacy mediate the relationship between physical activity and mental health. (3) stress self-management mediate the relationship between physical activity and mental health. (4) self-efficacy and stress self-management play a chain mediation role between physical activity and mental health.

## Materials and methods

### Participants and procedures

In March 2023, a cross-sectional study was conducted using a convenient sampling method to collect data from 400 middle school students in Jinan, Shandong province. All methods were performed in accordance with the Declaration of Helsinki-Ethical Principles for Medical Research Involving Human Subjects and other relevant laws, regulations and ethical norms. Prior to the investigation, all participating investigators underwent formal training to ensure the study’s integrity. Students interested in participating were provided with an explanation of the study’s concept and purpose by the investigators. Upon agreeing to participate, students were given access to an electronic questionnaire, which they completed diligently. The questionnaire covered various aspects, including basic sociodemographic information, levels of physical activity, self-efficacy, stress self-management, and mental health indicators. I have followed the STROBE checklist of cross-sectional studies. The study protocol received ethical approval from the Ethics Committee of the School of Basic Medical Sciences, Shandong University (Approval No. ECSBMSSDU2023-1-74).

400 participants completed the questionnaire, and 43 of them were excluded due to miss values with an effective rate of 89.25%. Among them, 198 (55.5%) were male and 159 (44.5%) were female, 140 (39.2%) were grade 1, 98 (27.5%) were grade 2, 119 (33.3%) were grade 3, 17 (4.8%) came from single-parent family, all came from city. The average age of participants was 13.74 (SD = 1.037) from age 12–16 years.

### Measures

#### Physical activity

The Health Promotion Lifestyle Profile includes Six dimensions that describe self-initiated actions and perceptions of health promotion behaviours^34^. In this study, we used physical activity dimension that include eight items to evaluate how often they adopt promotion physical activity. A 4-point Likert scale was used for quantification, with a score of 1 (never) to 4 (routinely) for each item. The scoring interval is 8 to 32 points. Higher scores indicated better exercise habits. In current study, Cronbach’s alpha coefficient is 0.9.

#### Self-efficacy

The Chinese version of the General Self-Efficacy Scale revised by Wang et al. was used to measure global self-efficacy of adolescents. The GSES consists of 10 items with 4-point score ranging from 1 (not at all true) to 4 (completely true)^35^. The range of the total scale was 10–40 points. Higher scores indicated higher levels of general self-efficacy. In this study, the Cronbach’s alpha coefficient is 0.92.

#### Stress self-management

The Health Promotion Lifestyle Profile (HPLP) stress management subscale was used to measure the adolescent stress self-management level^34^. The questionnaire on stress self-management includes 8 items and asked respondents to indicate how often they adopted health-promoting stress management behaviours. A 4-point Likert scale ranging from 1 (never) to 4 (routinely) was used to quantification. The range of total scale was 8–32 points. Higher the scores indicated higher levels of stress self-management. The Cronbach’s alpha coefficient is 0.87.

#### Mental health

The Kessler 10 Psychological Distress Scale (K10) is a short self-management rating scale that can detect the risk of psychological conditions in a population^36^. The 10-item scale measured the frequency of non-specific mental health-related symptoms such as anxiety and depression levels experienced in the previous 4 weeks. Likert’s 5-point scoring method was used for each question, and 1 (all the time) to 5 (hardly) points were scored. Higher the scores indicated better mental health. The Cronbach’s alpha coefficient is 0.95.

### Statistical analysis

The data initially collected through the questionnaire platform, specifically the Questionnaire Star platform (https://www.wjx.cn/), was exported for analysis. Descriptive statistics, Cronbach’s alpha test, reliability assessments, and spearman correlation analyses were conducted using SPSS version 27. For the mediation analysis, Hayes’ PROCESS macro in SPSS (version 4.1) was employed. The bootstrap method, involving 5000 resampling iterations to establish robustness and accuracy, was utilized to establish 95% confidence intervals (CIs) for determining the significance of mediating effects. Significance was attributed to direct or indirect effects when the CI did not encompass zero. Gender, age, grade and family structure were included as control variables in the model.ALL variables were standardized before their inclusion in the mediation model.

### Institutional review board statement

All materials and procedures of this study were approved by the Ethics Committee of the School of Basic Medical Sciences, Shandong University (Approval No. ECSBMSSDU2023-1-74).

### Informed consent statement

Written informed consent has been obtained from the patients.

## Results

### Common method bias analysis

The data for this study were gathered through an online self-assessment approach, which has the potential to introduce common method bias. To address this concern, Harman’s one-way analysis of variance test was conducted to scrutinise the factors associated with all the items encompassed in the study. Through exploratory factor analysis, 7 factors emerged with eigenvalues surpassing 1. However, the variance explained by the first factor was 30.06%, which is below the critical threshold of 40%. This analysis suggests that there is no significant common method bias^37^.

### Descriptive statistics and correlation analysis

Descriptive statistics of the study variables and their bivariate correlations are shown in Table 1. When r ≥ 0.4, there is a moderate and strong correlation^38^. The results indicated that physical activity was significantly and positively associated with self-efficacy (r = 0.409, p < 0.01), stress self-management (r = 0.716, p < 0.01) and mental health (r = 0.224, p < 0.01). Self-efficacy (r = 0.256, p < 0.01) was positively and significantly related to stress self-management (r = 0.406, p < 0.01) and mental health. In addition, there was a positive and significant association between self-efficacy and stress self-management (r = 0.373, p < 0.01).

**Table 1:** Descriptive analysis and bivariate correlations among key variables.

	M	SD	1	2	3	4
Physical activity	20.54	20.182	1	0.409400**	0.716677**	0.223283**
Self-efficacy	29.46	4.859		1	0.406379**	0.256300**
Stress self-management	21.4	4.616			1	0.373386**
Mental health	40.59	8.763				1

**p < 0.01.

### Multicollinearity test

Since there was a significant correlation among all variables, multicollinearity problem may existed leading to unstable results. Therefore, this study conducted multicollinearity diagnostics and standardized the predictor variables in each subsequent equation (to Z-scores). The results found that the tolerance (1–17.7) of all predictor variables is greater than 0.1, and the Variance Inflation Factor (1.240–2.128) is less than 5. Therefore, there is no serious multicollinearity problem in the data, which meets the conditions for further chain mediation analyses.

### Mediation analyses

After controlled variables such as gender, age, grade, and family structure among adolescents, a mediation effect test procedure was employed to assess the indirect impact of physical activity on mental health. This indirect effect was found to be mediated by self-efficacy and stress self-management^39^. The model’s fit and the significance of each path coefficient were evaluated using the PROCESS macro program in SPSS, as outlined by Hayes (2017).

Table 2 shows the regression coefficients for the self-efficacy and stress self-management mediators. The results showed that physical activity was positively and significantly associated with self-efficacy (β = 0.2797, p < 0.001) and stress self-management (β = 0.5535, p < 0.001). Self-efficacy and stress self-management (β = 0.1545, p < 0.001) and mental health (β = 0.2858, p < 0.001) was positively and significantly related. In addition, stress self-management and mental health was positively and significantly related (β = 0.6188, p < 0.001). No significant association was observed between physical activity and mental health.

**Table 2:** The results of the regression estimate of the chained mediation model.

Outcome variable	Model 1 Self-efficacy			Model 2 Stress self-management			Model 3 Mental health		
	β	SE	t	β	SE	t	β	SE	t
Physical activity	0.2797	0.0361	7.7433***	0.5535	0.035	15.7951***	− 0.1785	0.0926	− 1.9265
Self-efficacy				0.1545	0.0479	3.2284***	0.2858	0.0981	2.9131***
Stress self-management							0.6188	0.108	5.7304***
R2	0.4275			0.7317			0.42434		
F	15.7017			67.2043			10.9481		

***p < 0.001.

**Figure 2:**
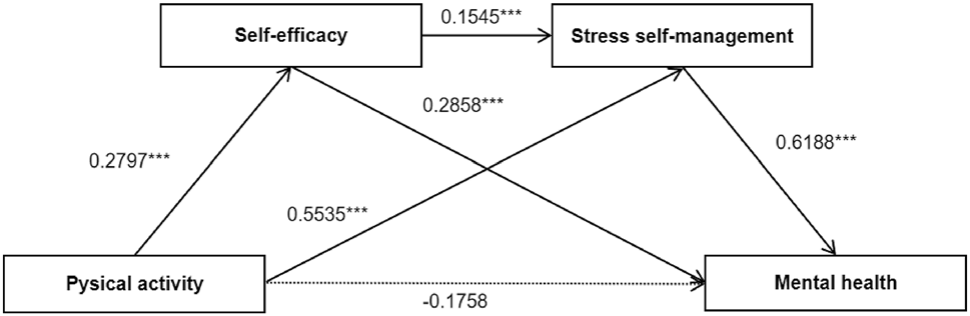
Effect of the chain-mediating model of self-efficacy and stress self-management on physical activity and mental health. ***p＜0.001.

Table 3 and Fig. 2 illustrates the results of the mediation analysis. The direct effect of physical activity on mental health was not significant, but the indirect effect was significant (95% CI: 0.3061-0.6065). The indirect effects of physical activity on mental health via self-efficacy and stress self-management were 0.0799 (95% CI: 0.0041-0.1801) and 0.3425 (95% CI: 0.2044-0.4948) significantly. In addition, the chained mediating effect of self-efficacy and stress self-management was 0.0267 (95% CI: 0.0070-0.0560). Therefore, self-efficacy and stress self-management play a complete mediating role between physical activity and mental health.

**Table 3:** Results of mediation analysis.

Effect	Pathways	Estimated	Boot S.E	95% C.I	
				Lower	Upper
Direct effect	PA → MH	− 0.1785	0.0962	− 0.3607	0.0037
Effect	PA → SE → MH	0.0799	0.0459	0.0041	0.1801
	PA → SSM → MH	0.3425	0.0732	0.2044	0.4948
	PA → SE → SSM → MH	0.0267	0.0126	0.0070	0.0560
Total effect		0.2707	0.0696	0.1338	0.4076
Total indirect effect		0.4492	0.0752	0.3061	0.6065

***P < 0.001. *S.E* standard error, *CI* confidence interval. *PA* physical activity, *MH* mental health, *SE* self-efficacy, *SSM* stress self-management.

## Discussion

This study explored the underlying mechanisms that link physical activity, self-efficacy, stress self-management, and mental health. The findings affirmed a noteworthy positive correlation between physical activity and mental health, thereby substantiating hypothesis H1. These results align with the findings of Doré et al. and consequently provide additional confirmation that physical activity stands as a crucial protective factor for mental health^7^.

Simultaneously, this study revealed that the link between physical activity and mental health lost its significance once the two mediating variables of self-efficacy and stress self-management were introduced. Moreover, the comprehensive indirect effect of these two mediating variables was deemed significant, and the indirect effect significantly outweighed the direct effect. This pattern indicated that self-efficacy and stress self-management jointly mediated the connection between physical activity and mental health. The outcomes indicated that physical activity initially predicted mental health without accounting for mediating variables. However, when self-efficacy and stress self-management were considered as mediating variables, their presence mediated the relationship between physical activity and mental health. This suggests that physical activity serves as a more distal factor influencing mental health, potentially due to its intrinsic nature. Physical activity is a consciously planned behavior rooted in personal intentions and motivations^40^. It also constitutes a behaviour shaped by external environmental influences, serving personal development or external incentives^41^. Positive outcomes or experiences gained from physical activity, particularly when proportional gains and positive experiences are achieved, are more likely to yield enhanced effects on mental health^42–44^. When adolescents were unable to obtain a good experience from physical activity or fail to meet their motivations, it may not have an impact on their mental health^45^. Physical activity experiences varied among individuals, and the effects of physical activity are not immediately perceptible^46,47^. Mental health improvements required prolonged intervention and was influenced by a multitude of factors^48^, with physical activity being just one of them. The time lag in observing the effects of physical activity on mental health can lead to its influence being overshadowed or affected by other factors, such as stressors and unexpected events in life^49^. Therefore, making adolescents more promptly aware of the benefits of physical activity is a core element in promoting their mental health.

The findings further substantiate the assertion that physical activity can influence mental health via the autonomous mediation of self-efficacy and stress self-management, as well as through the interconnected mediation of both. Hypotheses H2, H3, and H4 were confirmed. This underscores the significance of bolstered self-efficacy and proficient stress self-management as fundamental routes through which physical activity exerts its impact on mental health. In contemporary times, the Resilience Portfolio Model has emerged as a vital framework for comprehending the holistic landscape of protective factors and mental health processes among individuals navigating adversity^50–52^. This model delineates protective factors into three domains: regulation, meaning-making, and interpersonal factors, highlighting the pivotal role of enhancing self-worth, refining self-regulation, and fostering positive interpersonal relationships as crucial protective mechanisms for mental health^44^. Previous studies have illuminated the mediating role of resilience in connecting physical activity with mental health^53^. Likewise, research has identified self-efficacy and stress self-management, integral components of personal value and self-regulation, respectively as correlates of regular physical activity^32,33^. Self-efficacy theory posits that self-efficacy levels correlate with the perception of one’s capabilities and the mobilization of intrinsic cognitive resources, significantly influencing coping efficacy. Individuals endowed with high self-efficacy are inclined to harness their internal resources to confront life’s changes, thus showcasing enhanced stress management prowess. Conversely, individuals with lower self-efficacy often perceive themselves as incapable of effectively managing various life stressors, which may lead to passive coping strategies and reduced stress management efficacy. Further investigations have highlighted the association between self-efficacy and the behavioral stages of stress management, as outlined by the transtheoretical model^54^. The decrement in self-efficacy coincides with diminished engagement in stress self-management behaviors. Consequently, the impact of physical activity on mental health is channeled through the pathways of personal value and self-regulation, as well as through the intricate chain mediation involving personal value and self-regulation.

Indeed, the mediating role of self-efficacy can be illuminated from two key perspectives, both centered on the reinforcement of the personal value of physical activity. From this vantage point, self-efficacy operates through the lenses of individual perceptions regarding the effects and experiences derived from engaging in physical activity. Physical activity yields direct associations with a host of favorable outcomes encompassing physical fitness, body image, subjective physical well-being, positive emotional experiences, self-esteem, and overall life satisfaction^42^. This amalgamation of factors collectively constitutes a cognitive resource reservoir that bolsters mental health and fosters positive well-being. Those individuals who experience positive outcomes and emotions stemming from physical activity are more inclined to consistently take part in it. This sustained engagement fosters heightened self-perceptions, thereby contributing to elevated self-efficacy. As adolescents’ self-efficacy gains traction, the net result is a mitigation of negative emotions and a more optimistic approach to confronting life’s challenges. Previous research has underscored that mental health has direct links to positive and negative memory biases and positive interpretation biases, while the relationship with negative interpretation biases remains non-significant^55^. Consequently, individuals who reap positive exercise experiences and outcomes through physical activity tend to manifest heightened self-efficacy. This self-efficacy equips them with the tools to confront life’s stressors with a more optimistic disposition, thereby cultivating a more favorable and resilient psychological well-being.

From an alternative standpoint, the mediation effect of stress self-management can be illuminated through the lens of augmented self-regulation. A multitude of studies have underscored that physical activity engenders positive emotional experiences and exhibits a positive correlation with favorable responses to external stimuli^12,27^. When confronted with stress-inducing stimuli, individuals who engage in regular physical activity are more inclined to opt for adaptive coping mechanisms. Rooted in prior research, heightened levels of habitual physical activity are linked to an elevated capacity for stress self-management, for two plausible rationales. Firstly, individuals who consistently partake in physical activity tend to perceive lower levels of stress^24^. This, in turn, translates into a milder stress response. Such individuals are apt at emotional regulation, consequently manifesting a greater self-perceived efficacy in managing stress. Secondly, physical activity constitutes a deliberate behavior driven by intentions and goals, necessitating the coordinated functioning of an individual’s cognitive and executive systems. The self-regulatory system, meanwhile, is built on an individual’s cognitive response to intricate external contexts. Individuals who routinely engage in physical activity generally engage in positive cognitive reappraisal, enabling them to navigate changes in their external environment through adaptive means^27^. This skill set is indicative of heightened stress self-management capability.

In conclusion, this study holds noteworthy significance on multiple fronts. Theoretically, it constructs a comprehensive chain mediation model, unraveling potential mechanisms through which physical activity influences mental health. This contribution bears substantial theoretical implications for understanding the intricate underpinnings of mental health outcomes. Moreover, this research represents the pioneering exploration of the interplay between physical activity, self-efficacy, stress self-management, and mental health within the context of Chinese adolescents, thereby extending the application of The Resilience Portfolio Model. From a practical standpoint, this study offers valuable guidance for fostering the mental health of adolescents.

The outcomes of this study provide actionable insights into enhancing adolescent mental health. First, educational bodies, schools, and parents should prioritize the cultivation of adolescents’ awareness and habits related to physical activity. This strategic emphasis can effectively mitigate negative emotions through exercise, thereby nurturing robust mental health among adolescents. Furthermore, educational settings, especially physical education, should concentrate on instructing students to set goals for their physical activity endeavors. Conducting assessments of exercise objectives and outcomes, accompanied by timely encouragement, can notably bolster adolescents’ self-efficacy. Finally, schools were advised to disseminate information on the positive influence of consistent participation in physical activities on the enhancement of stress self-management aptitude and mental well-being. Schools can proactively broaden the scope of physical activity offerings that align with adolescents’ physiological attributes and preferences, thereby providing multifaceted stress-relief avenues and facilitating the ongoing improvement of mental health.

## Limitation and implications

Despite the valuable insights provided by this study, it is important to acknowledge its inherent limitations. First and foremost, the study design employed was cross-sectional in nature, thus precluded the establishment of causal relationships. To establish causal inferences, future research endeavors could incorporate experimental methodologies or longitudinal follow-up investigations. Another limitation stems from the questionnaire-based approach utilized in this study, with subjects self-reporting their experiences. This method introduces the potential for self-report bias, subsequently impacting the accuracy and reliability of the gathered data. In subsequent studies, a combination of questionnaire and interview methods might yield more comprehensive and accurate measurements of the variables under investigation. Furthermore, the study’s focus on the mediating role of self-efficacy and stress self-management leaves room for the inclusion of additional relevant moderating variables in future research. By incorporating these variables, a more comprehensive understanding of how physical activity influences mental health can be achieved, allowing for a more nuanced exploration of the underlying dynamics.

In summary, this study elucidated the intricate relationship between physical activity and mental health, revealing the mediating roles of self-efficacy and stress self-management. This study underscores that the impact of physical activity on adolescents’ mental health operates through independent mediation of self-efficacy, independent mediation of stress self-management, and chain mediation involving both self-efficacy and stress self-management. These findings enrich our understanding of the predictive influence of physical activity on mental health. The implications of these findings extend to interventions aimed at fostering physical activity engagement, enhancing self-efficacy, and promoting effective stress self-management, all of which have the potential to positively contribute to enhancing adolescents’ mental health. As the field advances, future research endeavours could delve deeper into discerning which specific types of physical activity are particularly effective in bolstering self-efficacy and stress self-management abilities. This could provide valuable insights into crafting targeted and efficacious interventions to improve adolescent mental health.

## Data Availability

The data supporting this study’s findings are available from the corresponding author upon reasonable request.
